# Cholesterol metabolism in neurodegenerative diseases: mechanisms and therapeutic advances

**DOI:** 10.1186/s13024-026-00951-3

**Published:** 2026-05-28

**Authors:** Dezhen Tu, Yuxiao Zheng, Pingping Ding, Miao Shen, Wangyang Tang, Bin Wei, Hongyan Wang

**Affiliations:** 1 Innovative Institute of Tumor Immunity and Medicine, Anhui Province Key Laboratory of Tumor Immune Microenvironment and Immunotherapy, The First Affiliated Hospital of Anhui Medical University, Hefei, 230022 China; 2 State Key Laboratory of Cell Biology & Key Laboratory of RNA Science and Engineering, CAS Center for Excellence in Molecular Cell Science, Shanghai Institute of Biochemistry and Cell Biology, University of Chinese Academy of Sciences, Chinese Academy of Sciences, Shanghai, 200031 China; 3 Key Laboratory of Systems Health Science of Zhejiang Province, School of Life Science, Hangzhou Institute for Advanced Study, University of Chinese Academy of Sciences, Hangzhou, 310024 China; 4 Institute for Translational Brain Research, State Key Laboratory of Medical Neurobiology, MOE Frontiers Center for Brain Science, Fudan University, Shanghai, 200032 China

**Keywords:** Cholesterol metabolism, Neurodegeneration, Microglia, Neuron, Astrocyte, Oligodendrocyte

## Abstract

Cholesterol metabolites are abundant in the central nervous system (CNS) that regulate cell membrane fluidity, signal transduction, and inter- and intracellular vesicular transport, as well as cell proliferation/cell death or migration. Brain cholesterol synthesis and metabolism are tightly coupled to the functional homeostasis of neurons, glial cells or microglia, and dysregulation of these processes has been strongly implicated in neurodegenerative diseases such as Alzheimer’s disease (AD), Parkinson’s disease (PD), and Huntington’s disease (HD). This review provides a comprehensive overview of how cholesterol synthesis, esterification, efflux, uptake, and oxidation affect the CNS function, highlighting the function of key enzymes or metabolites in distinct brain cell types during neurodegeneration. Based on single-cell/nucleus RNA sequencing data from the brains of AD, PD, and HD patients, we summarize cell-type-specific genes in cholesterol metabolism pathways, shedding new light to understand cellular heterogeneity. The role of cholesterol-derived neurosteroids in neurodegenerative diseases is also discussed. Furthermore, how cholesterol metabolites modulate the formation, aggregation, and degradation of amyloid-β (Aβ), α-synuclein and huntingtin, as well as Tau protein phosphorylation are outlined. Finally, future research directions are proposed that aim to understand neurodegenerative diseases with new angle.

## Introduction

Cholesterol is the primary steroidal compound in mammals and widely distributed in animals. Particularly, cholesterol is abundant in the CNS, and the brain contains over 20% of the body’s total cholesterol [1]. As an essential component of cell membranes, cholesterol contributes to maintaining membrane fluidity, signaling transduction, intracellular and intercellular vesicular transport [2]. Cholesterol can generate steroid hormones, vitamin D and other key metabolites, which play essential roles in fundamental cellular activities, including cell proliferation, cell death or migration [3, 4]. In the brain, cholesterol metabolism is closely regulated to maintain functional homeostasis of neurons or glial cells. Cholesterol promotes synaptic vesicles fusion with presynaptic membranes, mediates neurotransmitter release, alters the kinetics of postsynaptic membrane receptors, and influences synaptic activity. Mature neurons possess limited capacity for cholesterol synthesis under physiological conditions, relying on exogenous cholesterol to maintain their functions [1]. In contrast, astrocytes efficiently synthesize cholesterol from acetyl-CoA, and supply cholesterol to surrounding neurons to support neuronal function and memory formation [2]. Oligodendrocytes also require large quantities of cholesterol to construct myelin sheath, which is critical for rapid and efficient nerve conduction [5]. Moreover, cholesterol metabolism influences immune cell function and inflammation. In the brain, microglia are the resident macrophages, which can phagocytosis cell debris and clear excess cholesterol [6].

Cholesterol metabolism is a complex process encompassing synthesis, storage, efflux, transport, intake, esterification, oxidation, and biotransformation, which involves diverse enzymes and metabolic intermediates. Advanced reports have identified how some key enzymes regulate cellular function, including sterol regulatory element-binding proteins (SREBPs) as transcription regulators; 3-hydroxy-3-methylglutaryl-CoA reductase (HMGCR) in the synthesis of mevalonic acid (MVA); 7-Dehydrocholesterol Reductase (DHCR7) in the final steps of cholesterol synthesis; and sterol O-acyltransferase 1 (SOAT1) for converting free cholesterol to cholesterol esters. Major metabolic intermediates include MVA, influencing cell signaling and protein modification; lanosterol, regulating membrane fluidity and stability; squalene, involving in cell membrane construction and repair; 7-dehydrocholesterol, promoting type I IFN production and inhibiting ferroptosis.

Under physiological homeostasis, synthetic enzymes are primarily expressed in astrocytes, followed by oligodendrocytes, and are present at much low levels in neurons and microglia. Nevertheless, certain cholesterol-metabolizing enzymes exhibit cell-type specific expression patterns. For example, neurons highly express cholesterol 24-hydroxylase (CYP46A1) that converts cholesterol into 24-hydroxycholesterol (24-OHC) and acyl-CoA; cholesterol acyltransferase 1 (ACAT1) that esterifies cholesterol. In contrast, microglia express high levels of TREM2 (triggering receptors expressed on myeloid cells 2) that regulates lipid intake [7], and cholesterol 25-hydroxylase (CH25H) that produces 25-hydroxycholesterol (25-OHC). Interestingly, the expression and activity of cholesterol-metabolizing enzymes undergo significant changes during pathological neurological diseases. Cholesterol dysregulation is closely associated with various neurodegenerative diseases, such as AD, PD, and HD. While insufficient cholesterol can impair memory formation, excessive cholesterol accumulation can disrupt synaptic plasticity and induce apoptotic neuronal death.

In this review, we overview multiple processes of cholesterol metabolism in the CNS, and explore the roles of key enzymes and metabolites. We also summarize highly expressed cholesterol metabolism enzymes in different cell types from the brains of AD, PD, and HD patients. The regulatory function and mechanism of cholesterol metabolites to affect the formation, aggregation, and degradation of Aβ, α-synuclein and huntingtin, as well as the phosphorylation of Tau protein are discussed. These findings help us to understand neurodegeneration from a new angle.

## Brain cell type-specific cholesterol metabolism in neurodegeneration

**Fig. 1:**
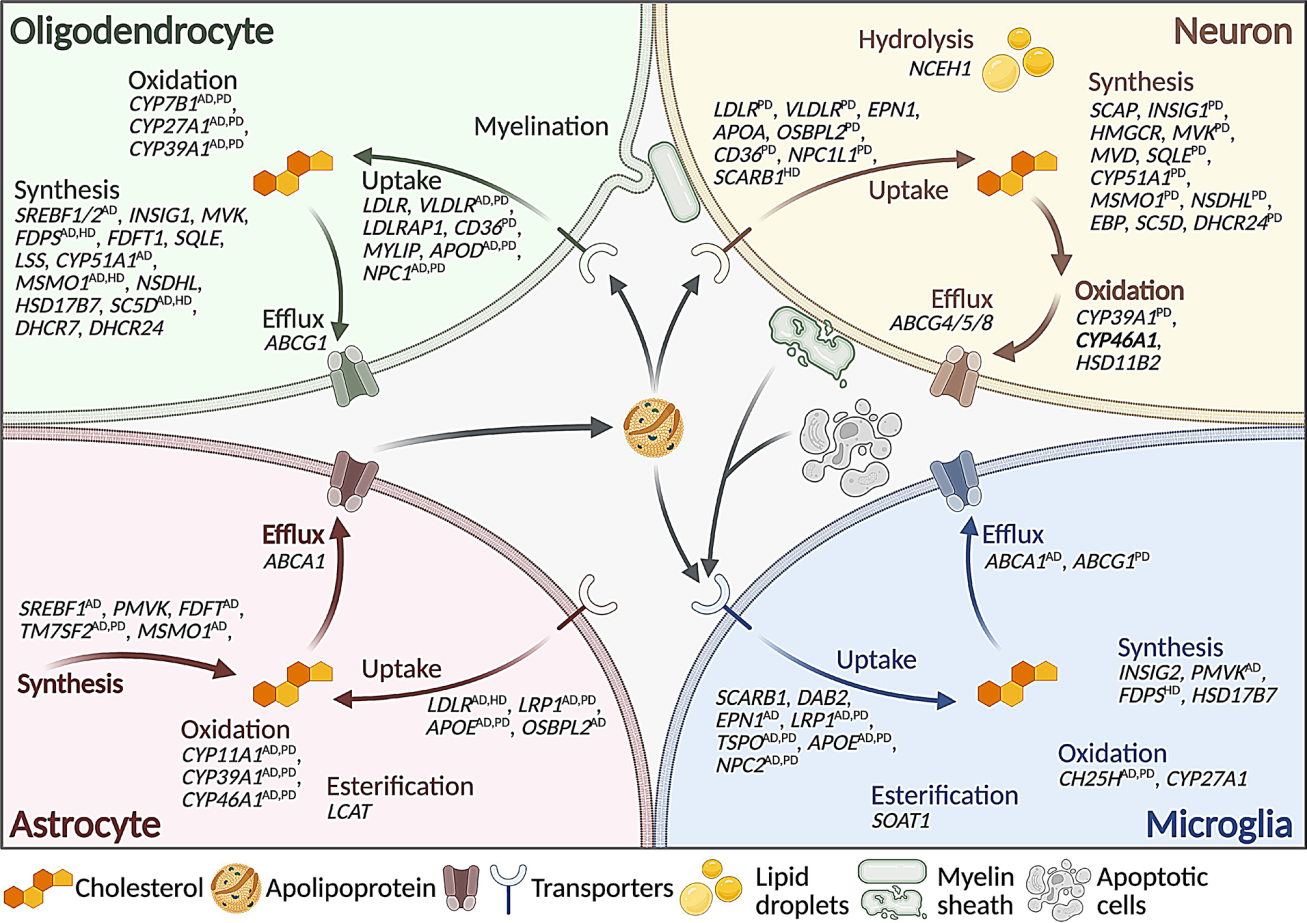
Genes highly expressed in various cholesterol metabolic processes in the brains of AD and PD patients. The intracellular cholesterol pool is sustained through a balance of *de novo* biosynthesis, uptake, esterification, oxidation, and efflux. This figure integrates scRNA-seq and snRNA-seq data from multiple studies (AD: GSE157827, GSE174367, GSE222494, GSE254205; PD: GSE161045, GSE184950, GSE193688, GSE243639; HD: GSE180928, GSE242197), and shows highly expressed genes in AD, PD, or HD (superscripts indicate genes upregulated in AD, PD, or HD respectively, while those without superscripts are upregulated in both). Astrocytes, the brain’s main cholesterol producers, release it via lipoproteins for uptake by neurons, microglia, and oligodendrocytes. Neurons take up cholesterol, oxidize it to oxysterols, and mediate efflux, driving brain cholesterol recycling. Microglia maintain homeostasis by phagocytosing damaged myelin and apoptotic cells, reusing their lipids/cholesterol. Oligodendrocytes need cholesterol for myelin synthesis. Created in https://BioRender.com

The brain is isolated from the peripheral cholesterol sources by the blood-brain barrier (BBB), which prevents the influx of circulating cholesterol into the CNS [8]. Nearly all brain cholesterol is synthesized locally via de novo pathways, which is tightly regulated in a cell type- and developmental stage-specific manner [9]. During early CNS development, neurons exhibit high cholesterol biosynthesis activity to support neuron growth, differentiation, and synaptogenesis-processes [2]. Oligodendrocytes maintain high cholesterol synthesis levels to form myelin sheaths that tightly enclose neuronal axons and dendrites, ensuring rapid and efficient neural signal conduction [10]. This elevated cholesterol production in neurons and oligodendrocytes is crucial for proper CNS development and function. As individuals mature into adulthood, the cholesterol metabolic landscape of CNS shifts. Mature neurons reduce their intrinsic cholesterol synthesis and become increasingly reliant on astrocyte-derived cholesterol. Astrocytes are characterized by their unique metabolic profiles and extensive cellular connections, playing a central role in supplying cholesterol in the adult CNS [11]. In contrast, microglia exhibit a distinct profile of cholesterol utilization. In resting microglia, cholesterol demand is low. In response to pathological stimuli such as injury, infection, or aging, microglia are rapidly activated and engage in phagocytosis or inflammatory response, with markedly increased demand for cholesterol [12]. By analyzing single-cell/single-nucleus RNA sequencing (sc/snRNA-seq) data, we identify cell-type-specific high-expression genes related to cholesterol metabolism from the brains of AD, PD, and HD patients, revealing distinct cellular heterogeneity in cholesterol dysregulation (Fig. 1).

### Cholesterol biosynthesis

Cholesterol biosynthesis in the brain is a tightly regulated process that occurs predominantly in the cytoplasm and endoplasmic reticulum (ER). It involves over 20 enzymatic steps, starting from acetyl-CoA and proceeding through three major stages: the synthesis of mevalonate, squalene, and ultimately cholesterol. Three key rate-limiting enzymes include HMGCR, squalene synthase (SQS), and lanosterol synthase (LSS). Initially, HMGCR catalyzes the conversion of HMG-CoA to mevalonate, a critical rate-limiting step. Mevalonate undergoes a sequence of phosphorylation and decarboxylation reactions to produce isopentenyl pyrophosphate (IPP) and its isomer, dimethylallyl pyrophosphate (DMAPP). SQS condenses two molecules of farnesyl pyrophosphate (FPP) to generate squalene. These isoprenoid units are gradually condensed to form longer polyprenyl chains. Ultimately, under the action of lanosterol synthase, these chains are cyclized into lanosterol, which subsequently undergoes a series of complex modifications to yield cholesterol.

Statins (e.g., rosuvastatin, simvastatin) target HMGCR, widely used to lower peripheral cholesterol. In the brain, the activity of HMGCR is associated with neuronal aging. Treatment with simvastatin has been shown to alter aging phenotype in induced neural stem cell lines (iNSCs) and reduce neurotoxicity [13]. Additionally, simvastatin functions as a ligand for peroxisome proliferator-activated receptor alpha (PPARα), enhancing the expression of brain-derived neurotrophic factor (BDNF) via activating PPARα-mediated cAMP response element-binding protein (CREB) [14]. However, statins can impair synaptic function similar to γ-secretase inhibition [15]. Genetic polymorphisms in the *HMGCR* (e.g., rs17244841, rs3846662, rs17238540) are associated with cognitive decline in PD Patients [16]. Notably, lovastatin reduces severity of L-DOPA-induced dyskinesia in the 6-hydroxydopamine (6-OHDA) rat PD model [17]. The *HMGCR* rs3846662 variant influences the AD onset and progression [18]. It is worth mentioning that lowered LDL-cholesterol levels via *HMGCR* variants does not causally increase the risk of AD or PD [19]. Squalene synthase inhibition via squalestatin protects against Aβ42-induced synaptic damage and neuronal death *in vitro* via suppressing cPLA2 activation and PGE2 production, not by altering Aβ42 uptake [20]. Lanosterol is reduced ~50% in the nigrostriatal pathway of 1-methyl-4-phenyl-1,2,3,6-tetrahydropyridine (MPTP)-treated mice, suggesting perturbed lanosterol metabolism in PD pathogenesis. Particularly, supplementation with exogenous lanosterol rescues dopaminergic neurons from 1-methyl-4-phenylpyridinium (MPP^+^)-induced cell death by inducing mild mitochondrial depolarization and promoting autophagy [21].

Cholesterol synthesis is tightly controlled by feedback inhibition, hormonal modulation, transcriptional control, and nutrient signaling. Hormonal regulation, such as by thyroxine, can stimulate the synthesis of HMGCR, thereby increasing cholesterol synthesis. Feeding-induced insulin and glucose surges activate HMG-CoA via the mTORC1–USP20-HMGCR signaling axis. At the transcriptional level, SREBPs serves as the key transcriptional regulatory factor. These ER-bound transcription factors sense intracellular cholesterol through the INSIG (Insulin-induced gene protein)-SREBP-SCAP (SREBP cleavage-activating protein) pathway. When intracellular cholesterol level is abundant, the SREBP-2/SCAP complex is anchored to the ER membrane via interactions between SCAP-SSD (sterol-sensing domain) and INSIG-1 or 2, blocking SREBP-2 activation and reducing further cholesterol absorption and synthesis. Conversely, when cholesterol levels decrease, this interaction is disrupted, allowing SCAP to expose its vesicular transport signal (MELADL), leading to the COPII-mediated vesicular trafficking of SCAP along with SREBP-2 from the ER to the Golgi. The complex is then cleaved by S1P and S2P, releasing the N-terminal transcription factor to enter the nucleus and activate cholesterol synthesis genes [22, 23]. Neuronal SREBP-1 activation is a crucial factor in N-methyl-D-aspartate glutamate receptor (NMDAR)-mediated excitotoxic neuronal death. The insig-1-derived interfering peptide (INDIP) not only prevents SREBP-1 activation, but also significantly reduces neuronal damage and improves behavioral outcomes [24]. In insulin-deficient diabetic mice, reduced expression of Srebp2 and its downstream genes in the hypothalamus leads to decreased brain cholesterol synthesis and synaptosome formation, reversible by intracerebroventricular injection of insulin [25]. Intracellular reactive oxygen species (ROS) can activate neuronal SREBP, leading to lipid droplet accumulation in glial cells, and the accumulated lipids undergo peroxidation in the presence of ROS. Reducing lipid droplet accumulation and peroxidation in glial cells alleviates neurodegeneration. Overexpression of lipases or reduction of ROS can significantly delay neurodegeneration [26]. Astrocytes-specific deletion of *Scap* reduces intracellular cholesterol levels and phospholipid secretion. Conditional knockout *Scap* mice increase immature synapses, but reduce presynaptic protein Snap-25 levels and synaptic vesicle numbers, leading to impaired presynaptic terminal development. Co-culture of primary astrocytes and neurons demonstrates that astrocyte-derived lipids can be absorbed by neurons to support synapse formation and function [27]. In the vertebrate nervous system, axonal myelination requires substantial lipids, and oligodendrocytes lipid synthesis is significantly supplemented by astrocytes. During embryogenesis, conditional *Scap* deletion in oligodendrocytes significantly delays myelin formation in the CNS [28]. In type II diabetic mice, microglia overexpress *Scap* to enhance the activity of Nlrp3 inflammasome in the Golgi, leading to increased pathological microglial responses and neuronal damage. Specific loss of *Scap* function in microglia alleviates inflammatory responses, defects of synaptic plasticity, and cognitive impairments [29].

In cellular HD models, mutant huntingtin (mHTT) protein binds to and sequesters the SREBP2/importin β complex in the cytoplasm, thereby preventing nuclear import of active SREBP2. This sequestration suppresses the transcription of cholesterol biosynthesis genes and reduces de novo cholesterol production [30, 31]. A targeted intervention study demonstrated that site-specific delivery of the N-terminal active fragment of SREBP2 to astrocytes in the R6/2 HD mouse model (a well-characterized model with robust mHTT overexpression) effectively activates the expression of cholesterol synthesis genes. Notably, this intervention significantly enhanced synaptic transmission, upregulated the transcriptional level of the dopamine receptor D2 (Drd2), and ameliorated behavioral deficits in HD mice. These results position SREBP2 dysfunction as a key contributor to cholesterol deficits in HD [32]. In a quinolinic acid-induced rat model of HD, simvastatin attenuates neuronal loss and reduces striatal lesion volume via upregulating the anti-apoptotic protein Bcl-2 and downregulating the pro-apoptotic protein Bax [33]. Several studies have shown that both atorvastatin and simvastatin ameliorated motor incoordination and memory impairment, while suppressing the expression of pro-inflammatory cytokines in a malonate-induced HD rat model [34]. Collectively, these findings demonstrate that statins exert neuroprotective effects using distinct HD models, likely through the synergistic integration of anti-apoptotic and anti-inflammatory mechanisms.

### Cholesterol esterification

Cholesterol esterification converts free cholesterol and fatty acids into cholesterol esters, representing one of the cholesterol storage mechanisms. Unlike peripheral tissues (e.g., liver and adipose), most brain cholesterol exists in a free form, and only a small fraction (≤1%) esterifies into cholesteryl esters that are sequestered in intracellular lipid droplets. Cholesterol esterification is catalyzed by sterol O-acyltransferase (ACAT, also known as SOAT) and lecithin cholesterol acyltransferase (LCAT). SOAT1 and SOAT2 are key genes involved in cholesterol esterification. SOAT1 is expressed in various brain cells, with the highest expression levels observed in microglia. Microglia scavenging activity leads to intracellular cholesterol accumulation, likely explaining the elevated expression of esterification-related genes. In neurodegenerative diseases and acute neuroinflammation, SOAT1 expression in microglia is upregulated. *Soat1* deficiency reduces lipid droplets and cholesterol crystals within microglia, along with increased expression of the cholesterol efflux-related gene *Abca1* [35, 36]. However, studies about how Soat1 regulates microglia inflammatory response are inconsistent. Some studies have shown that *Soat1* inhibition suppresses pro-inflammatory responses induced by LPS or excess cholesterol [37]. In demyelinating diseases, however, deletion of *Soat1* exacerbates inflammation and inhibit myelin regeneration [35]. LCAT, in contrast, catalyzes the esterification of cholesterol at the surface of high-density lipoproteins (HDL), transferring fatty acids from lecithin to cholesterol to form cholesterol esters and lysolecithin. LCAT is primarily expressed in astrocytes, with minimal expression in other cell types [38, 39]. However, it remains poorly defined about LCAT expression and function in neurodegenerative diseases.

Neutral cholesterol ester hydrolase (NCEH1) is a key enzyme that catalyzes the hydrolysis of cholesteryl esters into free cholesterol and fatty acids. In the brain, NCEH1 is predominantly expressed in neurons, providing free cholesterol precursors for the synthesis of synaptic membranes, neurotransmitters, and other components [40]. This function prevents the accumulation of lipid droplets that could impair neuronal function. Notably, NCEH1 has been shown to protect dopaminergic neurons from α-synuclein-dependent neurotoxicity, promote cholesterol-derived neurosteroid formation and lower cellular ROS in mitochondria [41].

In post-mortem brain tissues of HD patients, cholesteryl esters (CE) were specifically accumulated within core pathological regions, including caudate nucleus and putamen. The CE levels in the caudate nucleus and in the putamen were increased relative to controls. In contrast, no significant change of total CE concentration was observed in the cerebellum, a region less affected in HD, while several polyunsaturated CE subtypes (CE 18:2, CE 18:3, and CE 22:6) were significantly decreased. Despite the significant elevation of CE concentrations, protein expression of ACAT1 does not differ significantly in HD patients in either the caudate nucleus and putamen [42]. These findings suggest that CE accumulation may result from elevated ACAT1 enzyme activity rather than increased protein abundance, or alternatively, from impaired CE hydrolase function leading to delayed CE clearance.

### Cholesterol efflux

Cholesterol efflux can shift excess intracellular free cholesterol into the extracellular space via specialized transporter proteins, which is essential to preventing toxic intracellular cholesterol accumulation. In the brain, cholesterol efflux relies on the members of ATP-binding cassette (ABC) transporter family-transmembrane proteins that use ATP hydrolysis to actively transport various substrates (e.g., lipids, ions, metabolites) across the membrane. Among these, ABCA1 and ABCG1 are the principal transporters involved in cholesterol efflux in neurons and glial cells. ABCA1 is highly expressed in astrocytes and mediates the direct transfer of intracellular free cholesterol and phospholipids to extracellular apolipoproteins (e.g., APOA-I, a precursor of HDL), forming nascent HDL particles. By contrast, ABCG1, predominantly expressed in oligodendrocytes, directly transports intracellular free cholesterol to mature HDL particles, independent of APOA-I [43]. ABCG1 is considered more efficient than ABCA1 in this role. These two transporters act synergistically to regulate cholesterol efflux, and their expression is regulated by liver X receptor (LXR) activation.

In AD patients, decreased serum levels of ABCA1 is accompanied by elevated inflammatory cytokines, suggesting that ABCA1 dysfunction may synergize with neuroinflammation to promote disease progression [44, 45]. Loss-of-function of ABCA1 is associated with reduced plasma apolipoprotein E (APOE) levels and an increased risk of AD [46]. Dysfunction of ABCA1 leads to cholesterol accumulation, which activates beta-secretase 1 (BACE1) and enhances Aβ production [47]. In PDAPP*/ABCA1*-Transgenic mice (overexpressing ABCA1), cerebral Aβ deposition is significantly reduced, with a shift from amyloid plaques to non-fibrillar forms [48]. Additionally, ABCA1 agonists restore ApoE4-mediated lipid droplet formation and mitigate ROS-induced neurotoxicity, offering a novel therapeutic strategy for AD [49, 50]. In PD, Abca1 downregulation exacerbates α-synuclein aggregation into Lewy bodies. *Abca1* knockout mice exhibit increased dopaminergic neuron loss in the substantia nigra and aggravated α-synuclein aggregation, highlighting a neuroprotective role of *Abca1* in these neurons [51]. The complete *Abca1* knockout mice exhibit increased cortical neuronal loss, astroglial hyperplasia, impaired microglial phagocytosis, and enhanced tissue inflammation. Interestingly, neuron-specific conditional *Abca1* deletion leads primarily to astroglial hyperplasia without significant inflammation, whereas astrocyte-specific conditional deletion shows no apparent abnormalities [52]. Genetic polymorphism studies of *ABCA1* R219K show that AD patients with the RR genotype exhibit better cognitive function, while PD patients with the KK genotype could slow disease progression [44].

Using astrocytes derived from HD patients, researchers established an ABCA1 overexpression system and showed that conditioned medium (CM) from these astrocytes significantly enhance neurite outgrowth in HD patient-derived neurons. Conversely, ABCA1 knockdown in wild-type astrocytes abrogated the neurite-promoting activity of their CM on HD neurons [53]. These findings suggest ABCA1 as a critical regulator of HD neuronal growth and differentiation by mediating cholesterol-dependent crosstalk between astrocytes and neurons. Wild-type huntingtin (wtHTT) interacts with the ligand-binding domain (LBD) of LXR. Expansion of the CAG repeat tract in mHTT disrupts this interaction, weaking LXR-mediated transcription. In an HTT-knockout (HTT^−/−^) zebrafish, loss of wtHTT results in insufficient activation of LXR target genes involved in cholesterol efflux, and is accompanied by aberrant cartilage development. Notably, treatment with an LXR agonist partially rescues these early developmental defects and restores LXR target gene expression [54].

### Cholesterol uptake

Under the BBB barrier, peripheral lipoproteins carrying cholesterol are unable to enter the brain [10, 55]. Therefore, the *de novo* synthesis of cholesterol in the brain is critical, and requires significant energy expenditure. Astrocytes are significant contributors to cholesterol synthesis in the brain, supplying cholesterol to the brain microenvironment in the form of APOE-cholesterol complexes [56]. In contrast, neurons synthesize only a certain amount of cholesterol, and primarily rely on the uptake of lipoproteins from the extracellular environment, facilitating the flow and circulation of the cholesterol pool within the brain [57].

In astrocytes from the brains of *Apoe*-knockout AD mice, specific overexpression of human *APOE2*, *APOE3*, or *APOE4* induces the formation of fibrillary amyloid plaques and reduces soluble Aβ aggregates, independent of microglia. The overexpression also enables *APOE*-deficient microglia to exhibit clustering and a disease-associated microglia (DAM)-like response [58]. The late-onset AD risk gene *APOE4* disrupts neuronal functions, including mitochondrial OXPHOS, glucose metabolism, and synaptic density, by reducing the sequestration of fatty acids (FAs) in lipid droplets (LDs) [59, 60]. *APOE4* also decreases the clearance of neuronal FAs by astrocytes, leading to lipid accumulation in astrocytes and the hippocampus (Hipp). In contrast, APOE3 acts as an enhancer of neuronal lipid clearance [59]. In addition to APOE, in both in vitro and in vivo models of PD, the release of APOD from astrocytes significantly enhances survival of dopaminergic neurons, providing a new strategy for the treatment of PD [61].

Although neurons display a limited capacity for cholesterol synthesis, mature neurons are high-energy-consuming cells, exhibiting a rapid turnover of cholesterol at their projections and local demand exceeds their own synthetic capacity [62]. Consequently, mature neurons are more inclined to uptake cholesterol through LDLR and LRP1 (Low-density lipoprotein receptor-related protein-1) [63]. In SH-SY5Y neuroblastoma cells, overexpression of proprotein convertase subtilisin/kexin type 9 (PCSK9) significantly reduces the expression of LDLR and Apolipoprotein E receptor 2 (APOER2), which impairs cholesterol uptake and increases Aβ-induced neurotoxicity [64]. Genetic deletion of neuronal PCSK9 affects the synaptic localization of APOER2 and lipid droplets abundance, which ultimately impairs hippocampus-dependent cognitive function [65]. LRP1 serves as a key regulatory factor for the uptake of tau and α-synuclein in neurons; targeting LRP1 in neurons can decrease the propagation of tau and α-synuclein within the brain [66, 67]. The absence of neuronal LRP1 impairs the expression of insulin receptor β (IRβ), resulting in glucose intolerance that is also an early pathological event of AD [68, 69]. APOE particles derived from astrocytes supply cholesterol to neurons, which can be abolished by specific shRNA targeting LDLR or LRP1 on neurons [70].

Microglia can ingest cholesterol released from apoptotic cells or damaged myelin, which also take up lipoprotein particles derived from astrocytes [71]. APOE inhibits LPS-induced JNK activation through LDLR or LRP1 on microglia, thereby reducing inflammatory response [72]. LPS stimulates LRP1 shedding from microglia, and then soluble LRP1 amplifies neuroinflammation in microglia, converting an anti-inflammatory lipoprotein receptor into a pro-inflammatory product [73]. Overexpression of LDLR reduces the elevated expression of APOE in microglia of P301S tauopathy mice. Mice with ApoE deficiency and overexpressed LDLR harbor enlarged pools of oligodendrocyte progenitor cells (OPCs), showing greater preservation of myelin integrity under neurodegenerative conditions [74]. Low-density lipoprotein (LDL) stimulation inhibits clearance of myelin debris by microglia in vivo, leading to dysregulation of lipid metabolism and defects in supporting the regenerative properties of OPCs. However, microglia-specific knockdown of LDLR provides protective effects against ischemic demyelination [75]. TREM2 is specifically expressed on microglia and can sense lipids and regulate phagocytosis of myelin. In a cuprizone-induced demyelination model, *Trem2*-deficient microglia are unable to clear the myelin cholesterol they have ingested, leading to the accumulation of cholesterol esters within the cells to impair microglial function [76].

As key cells responsible for the formation and maintenance of myelin in the CNS, oligodendrocytes can synthesize a portion of their own cholesterol, and also utilize LRP1, VLDLR, and APOER2 to take up lipoproteins [5, 77]. During the myelin repair phase in demyelinating diseases, this uptake process may have been enhanced. In chronic cerebral ischemia, abnormal LDLR expression in oligodendrocytes disrupts myelination via intracellular signal transduction [78]. As the major genetic risk factor for AD, APOE4 is closely associated with declines in episodic memory [79]. APOE4 impairs myelination in the aging brain by blocking the transport of astrocyte-derived lipids to oligodendrocytes [80]. Lipid uptake receptors on oligodendrocytes can also take up lipid nanoparticles (LNPs), indicating remarkable potential for targeted gene expression to treat neurological disorders.

Cerebral cholesterol biosynthesis is significantly impaired in HD, and the expression of APOE in astrocytes is markedly reduced. This dual defect leads to a reduction in the cholesterol content of APOE-containing lipoproteins [81]. Notably, CM from ApoE-deficient primary astrocytes, as well as lipoprotein-depleted CM from wild-type astrocytes, fail to sustain neurite outgrowth and synaptic integrity in HD neurons [53]. These data highlight the indispensable role of APOE-mediated, lipoprotein-associated cholesterol to maintain the normal neuronal function. It suggests that therapeutic enhancement of astrocyte-mediated cholesterol homeostasis may ameliorate neuronal dysfunction in HD.

### Cholesterol oxidation

Cholesterol can be oxidized into various oxysterols. In general, the brain of mice contains relatively high levels of oxysterols, including 7α/7β-hydroxycholesterol (7α/7β-OHC), 7-ketocholesterol (7-KC), 24*S*-OHC, 25-OHC, and 27-OHC [82]. Cholesterol oxidation can be classified into two types. The first type involves oxidation mainly on the side chain of the cholesterol carbon skeleton, which is often catalyzed by specific enzymes. For example, the CH25H and CYP27A1 enzymes catalyze the conversion of cholesterol to 25-OHC and 27-OHC, respectively. The second type of oxidation, such as 7β-OHC and 5α,6α-epoxycholesterol (5α,6α-EC), occurs when ROS attacks the carbon skeleton ring of cholesterol, independent on catalytic enzyme [83, 84]. Notably, the same oxysterol can be generated via both enzymatic and non-enzymatic oxidation [85], such as 25-OHC.

Astrocytes synthesize a significant amount of cholesterol in the brain. Because of the BBB, excess cholesterol cannot be directly eliminated, which may pose a risk to brain health [86]. Neurons exhibit high expression of CYP46A1 to oxidize cholesterol to 24*S*-OHC, and 24*S*-OHC can easily cross the BBB, which is important for cholesterol turnover in the brain [87]. 24*S*-OHC is abundant in the brain and can regulate the occurrence and development of neurodegenerative diseases by influencing astrocytes, oligodendrocytes, and neurons [88]. Different from many oxysterols that are associated with neurological dysfunction and degeneration, 24*S*-OHC is believed to have neuroprotective effects. Overexpression or knockout of *Cyp46a1* respectively enhanced or impaired the learning abilities of mice [89, 90]. 24*S*-OHC can cause the ubiquitination and degradation of tau protein by upregulating the SIRT1/PGC1α/Nrf-2 axis, reducing neurotoxicity caused by tau protein accumulation [91]. The role of 24*S*-OHC also varies by sex. 24*S*-OHC can activate sex hormone signaling, including estrogen receptors, in neurons [92]. While previous studies have reported that cerebrospinal fluid (CSF) levels of 24S-OHC in patients with AD are positively correlated with both p-tau and t-tau [93, 94]. However, more recent evidence indicates that 24S-OHC is negatively correlated with neurodegenerative markers only in female patients. Consistent with these observations, overexpression of Cyp46a1 in female mice has been shown to improve cognitive function in aged mice, whereas aged-matched male mice exhibit anxiety-like behavior and memory impairment [92]. These apparently contradictory findings may be attributed to the larger sample size, the predominance of early-stage AD patients, sex-stratified analyses, as well as the exclusion of patients with comorbidities such as diabetes, hypercholesterolemia, and hypertension in the latter study. The anti-HIV drug efavirenz can activate Cyp46a1 at low doses, increasing the levels of 24*S*-OHC in plasma and CSF to improve cognitive abilities in 5×FAD mice [95]. On the other hand, high concentrations of 24*S*-OHC was reported to disrupt redox homeostasis and produce oxidative toxicity in neuroblastoma and glioma cell lines [96, 97]. Therefore, understanding 24*S*-OHC function in the nervous system need to consider its effective concentrations, and sex of the host.

27-OHC is produced mainly in the liver, which can cross the BBB to enter the brain to regulate myelination, lipid metabolism, and the development of neurodegenerative diseases [98]. 27-OHC promotes the cleavage of amyloid precursor protein (APP) into highly neurotoxic Aβ42 in SH-SY5Y cells, whereas 24-OHC does not affect the production of Aβ42 [99]. 27-OHC also interferes with the Th17/Treg balance, which may be associated with learning and memory impairments in mice [100]. 27-OHC can cause inflammatory damage to neurons or astrocytes through the TGF-β/NF-κB or TLR4/TGF-β signaling pathway, respectively [101]. Like 24*S*-OHC, 27-OHC is an endogenous selective estrogen receptor modulator [102]. In early AD-affected brain regions, such as the hippocampus, there is a distribution of estrogen receptors. 27-OHC regulates estrogen receptor signaling and loses estrogen-mediated neuroprotective functions, leading to neurodegeneration [103, 104]. 27-OHC can be metabolized downstream via CYP7B1. Both *Cyp27a1*-overexpressing and *Cyp7b1*-knockout mice accumulate 27-OHC, but *Cyp7b1*-knockout mice maintain intact learning and memory capacities [105, 106]. 27-OHC also exacerbates AD-related intestinal pathophysiology, particularly intestinal microbiota dysbiosis and intestinal barrier dysfunction [107].

In human AD brain tissue and in the brain tissues of transgenic mice carrying amyloid-β plaques or tau pathology, the expression of *CH25H* is significantly elevated [108]. DAM in AD mice show high expression of Ch25h, which oxidizes cholesterol to 25-OHC. When *Ch25h* is deleted and its product 25-OHC is reduced in PS19 mice that overexpress the human tau protein with the P301S mutation, age-related neurodegeneration and neuroinflammation are markedly reduced in the hippocampus, entorhinal cortex, and piriform cortex, accompanied by robust suppression of pro-inflammatory signals in microglia [109]. LPS can also increase *Ch25h* expression in microglia to further promote IL-1β production through caspase-1, and *Ch25h* knockout attenuates neuroinflammation [108]. Interestingly, the induction of 25-OHC in the brain by LPS is more pronounced in female mice, although no differences in *Ch25h* expression [110]; future investigation needs to ask whether this involves enzyme-independent induced 25-OHC production.

Cholesterol can be auto-oxidized to 7β-OHC under the action of ROS [111]. Stereotactic injection of 7β-OHC into the mouse hippocampus directly induces neuroinflammation accompanied by elevated levels of IL-1β and IL-6, enhances the amyloidogenic pathway, and impairs memory [112]. The brains of AD patients decrease 24*S*-OHC and increase levels of other oxysterols such as 25-OHC, 27-OHC, 7-KC, and 7α/7β-OHC, suggesting a possible key role of oxysterols in the pathogenesis of AD [112, 113].

Patients with PD have higher plasma levels of 27-OHC with lower levels of 24*S*-OHC [114, 115]. In aged mice, liver Cyp27a1 expression and plasma 27-OHC levels are increased. 27-OHC, but not 25-OHC, promotes α-synuclein aggregation; therefore Cyp27a1 deficiency can alleviate motor impairment in mice [114]. Although still no extensive research on how oxysterols affect the development of PD, changes of oxysterols have been identified in the brain or periphery from PD patients or mice.

CYP46A1 is significantly downregulated in HD patients and HD mouse models, as well as in mHTT-expressing cell lines. AAV (adeno-associated virus)-mediated restoration of CYP46A1 in the striatum markedly elevated the levels of 24S-OHC and normalizes the levels of cholesterol and lanosterol. These metabolic corrections correlate with improved motor function, reduced neuronal atrophy, and decreased mHTT protein aggregation [116]. Furthermore, 24S-OHC functions as an agonist of the N-methyl-D-aspartate (NMDA) receptor, and enhanced synaptic signaling may contribute to improved cognitive performance observed in HD following CYP46A1 delivery [117].

The research progress on oxysterols in various neurodegenerative diseases is summarized in Table 1.

**Table 1:** Oxysterols in neurodegenerative diseases

Oxysterols	Materials	Effect and Potential mechanism
24S-OHC	AD patients treated with low-dose efavirenz	elevated in plasma/CSF; allosteric CYP46A1 activation [95]
	SH-SY5Y	induces RIPK1 mediated necroptosis [96]
	*U*-87 MG	low dose: maintains redox homeostasis; high dose: increase oxidative stress [97]
	SK-N-BE	induces Tau degradation via SIRT1/PGC1α/Nrf2 axis [91]
	Cyp46a1 KO mice (male)	spatial/associative/motor learning deficits [90]
	Cyp46a1 OE mice (female)	brain/serum elevation improves spatial memory [118]
	Cyp46a1 OE mice (male)	anxiety-like behavior and worsened memory [92]
	Hipp AAV- Cyp46a1-injected THY-Tau22 mice (female)	rescues cognitive deficits, the impaired long-term depression, and spine defects [89]
	R6/2 mice	restores sterols; alleviates motor deficits; attenuates neuronal atrophy and protein aggregation [116]
27-OHC	SH-SY5Y	upregulates APP, BACE1, and Aβ42 [99, 101]
	WT mice (male)	impairs learning/memory; disturbs Th17/Treg immune balance-related immune responses [100]
	Cyp7b1 KO mice (male)	preserves learning, memory, neuronal morphology, and brain glucose uptake [119]
	Cyp27a1 OE mice (male)	cognitive decline, neuronal morphological impairment, and reduced glucose uptake [119]
	high-cholesterol diet rabbits (male)	elevation in hippocampus/serum exacerbates neurodegeneration via downregulation of ERα [104]
25-OHC	HMC3 microglia	increases IL-1β and MHC II expression [113]
	Ch25h KO microglia	attenuates IL-1β secretion and inflammasome/caspase-1 activation [108]
	Ch25h KO in PS19 mice (female)	reduces neuroinflammation, gliosis, tau aggregation, hippocampal/entorhinal cortex atrophy, and age-related neurodegeneration [109]
7β-OHC	WT mice (male)	intrahippocampally injected 7β-OHC induces memory/frontal/executive deficits; elevates TNF-α, IL-1β, IL-6 [112]

### Cholesterol-derived neurosteroids

Neurosteroids (NS) are steroid derivatives synthesized de novo primarily in the CNS (e.g., cerebral cortex, hippocampus) using cholesterol as a precursor. These molecules rapidly modulate the neuronal excitability and play critical roles in neural development, plasticity, and the maintenance of normal brain function [120–122]. According to their chemical structures, neurosteroids can be categorized into three main groups: pregnane neurosteroids, such as allopregnanolone (ALLO) and allotetrahydrocorticosterone (alloTHB); androstane neurosteroids, such as androstenediol (Adiol) and etiocholanone (Etio); and sulfated neurosteroids, such as pregnenolone sulfate (PREGS) and dehydroepiandrosterone sulfate (DHEAS) [122]. Functionally, pregnane and androstane neurosteroids mostly act as inhibitory neurosteroids, exerting sedative, anesthetic, anxiolytic, anticonvulsant, and neuroprotective effects by enhancing inhibitory signaling. In contrast, sulfated neurosteroids generally function as excitatory neurosteroids, increasing neuronal excitability and participating in learning and memory regulation; however, excessive concentrations may induce anxiety-like responses under pathological conditions [123, 124]. Unlike classical steroid hormones that primarily act through nuclear receptors to regulate gene transcription, neurosteroids exert rapid non-genomic effects, largely independent of intracellular steroid hormone receptors. Instead, they mainly function through allosteric modulation of ionotropic receptors, among which the bidirectional regulation of GABA-A receptors represents the most central and classic molecular mechanism [125–127]. Based on these unique molecular actions and physiological functions, neurosteroids have attracted increasing attention as potential therapeutic targets for multiple CNS disorders, including epilepsy, cognitive impairments, anxiety disorders, stress-related diseases, and depression.

Neurosteroids in AD. Multiple studies have reported reduced levels of key neurosteroids—including DHEAS, ALLO, and pregnenolone (PREG)—in several brain regions of patients with AD, such as the hippocampus, amygdala, frontal cortex, striatum, hypothalamus, and cerebellum. In particular, PREGS and DHEAS levels are significantly decreased in the striatum and cerebellum, while DHEAS concentrations are also markedly decreased in the hypothalamus. Serum DHEAS levels in AD patients are lower than in age-matched healthy individuals [128, 129]. Interestingly, cortical Aβ levels show a significant negative correlation with PREGS concentrations in the striatum and cerebellum, whereas cortical tau hyperphosphorylation is negatively associated with DHEAS levels in the hypothalamus [130]. These findings suggest that these neurosteroids may exert important neuroprotective effects during AD pathogenesis. Experimental studies further support this hypothesis. In male AD mouse models, exogenous administration of DHEAS significantly improves cognitive functions, particularly learning and memory [131]. In female 3xTg‑AD mice, combined treatment with progesterone (PROG) and estrogen reduces tau phosphorylation and ameliorates AD‑like neuropathological alterations [132]. Among the various dysregulated neurosteroids, ALLO represents one of the most promising candidates for clinical translation in AD therapy. Mechanistically, ALLO modulates the TLR4 signaling pathway by disrupting the interaction between TLR4 and MyD88, thereby inhibiting the downstream activation of NF‑κB and MAPK pathways and reducing the release of pro‑inflammatory cytokines such as TNF‑α, IL‑1β, and IL‑6, thus exerting anti‑inflammatory effects [133]. In addition, ALLO enhances the cerebral CX3CL1/CX3CR1 signaling and regulates astrocyte activation [134]. Notably, ALLO also shows therapeutic potential for depression comorbid with AD [135]. The randomized, double‑blind, placebo‑controlled Phase Ib/2a and Phase II clinical trial demonstrated that AD patients receiving ALLO treatment for 12 weeks exhibited a significantly slower rate of hippocampal atrophy compared with placebo-treated patients (ClinicalTrials.gov Identifier: NCT02221622, NCT04838301) [136–139]. In some APOE ε4 carriers hippocampal volume even increased. Furthermore, ALLO treatment improved the integrity of white matter microstructure, particulary in major fiber tracts such as the corpus callosum and thalamic radiations, and enhanced functional connectivity between AD‑vulnerable brain regions and the limbic system [136].

Neurosteroids in PD. In recent years, increasing evidence has highlighted the regulatory roles and therapeutic potential of neurosteroids in PD. Clinical studies have consistently demonstrated that circulating levels of ALLO and 5α-dihydroprogesterone (5α-DHP) are significantly reduced in both peripheral blood and CSF of PD patients compared with healthy controls, whereas PREG concentrations show a modest decreasing trend [140]. These findings indicate that endogenous inhibitory neurosteroid deficiency constitutes a pivotal biochemical feature of PD. Of particular clinical relevance, PD patients harboring pathogenic mutations in the glucocerebrosidase (GBA) gene exhibit even lower ALLO levels than non-mutated PD patients. Furthermore, ALLO concentrations are significantly negatively correlated with the severity of motor impairment, cognitive decline, and comorbid psychiatric manifestations, reinforcing a close relationship between neurosteroid dysregulation and PD disease severity [141]. Parallel alterations have been observed in experimental models. In 6-OHDA-induced rat model of nigrostriatal dopaminergic degeneration, striatal levels of PREG and 5α-DHP are significantly reduced, mirroring the neurosteroid alterations observed in human PD patients [142]. Together, these findings suggest that dysregulation of specific endogenous neurosteroids contributes to the initiation and progression of PD. Among these neurosteroids, ALLO, PREG, and PROG have emerged as particular promising candidates for therapeutic intervention. For example, exogenous ALLO administration effectively attenuates cognitive dysfunction in 6-OHDA-lesioned rats [143]. Additionally, ALLO exerts potent pro-proliferative effects on neural stem and progenitor cells, regulating the expression of cell-cycle-related genes and proteins in both rodent and human neural progenitors [144]. Based on these neuroregenerative properties, ALLO has advanced to Phase I clinical trials as a novel neurorestorative agent aimed at repairing damaged dopaminergic neuronal circuits and alleviating motor dysfunction in PD patients (ClinicalTrials.gov Identifier: NCT06263010). In the MPTP-induced mouse model of PD, early PROG intervention effectively modulates pathological neuroinflammation, suppresses excessive astrocyte activation, and preserves physiological levels of BDNF in the early disease stage [145]. Correspondingly, in 6-OHDA-induced rat PD models, PROG treatment not only mitigates motor deficits and cognitive impairment but also prevents the development of depression-like behaviors associated with parkinsonism [146]. In contrast, pregnenolone has shown efficacy in reducing L-DOPA-induced dyskinesia (LID), a major complication of long-term L-DOPA therapy. In PD rat models, pregnenolone administration dose-dependently attenuates LID without compromising the therapeutic efficacy of L-DOPA. Mechanistically, these effects are mediated mainly via reduced phosphorylation of DARPP-32 and ERK1/2 downstream of dopamine D1 receptors, diminished formation of D1–D3 receptor heteromers, and modulated BDNF expression in the striatum [147]. Notably, pregnenolone treatment also reduces LID in ovariectomized female parkinsonian macaques, further consolidating its high translational potential for clinical PD therapy [148].

Neurosteroids in HD. Compared with AD and PD, research on neurosteroids in HD remains relatively limited and largely exploratory. To date, no clear consensus has been reached regarding the systematic alterations of neurosteroids in the brain, peripheral blood of HD patients, or corresponding animal models. Existing clinical evidence mainly focuses on the correlation analysis of peripheral blood neurosteroids. One study demonstrated that plasma DHEAS levels decline with age in female HD patients, and patients with comorbid depression present lower plasma DHEAS levels than both non-depressed HD patients and healthy controls [149]. These findings suggest that the abnormal downregulation of DHEAS may be closely associated with the occurrence of neuropsychiatric symptoms in HD patients. In vitro cell culture experiments have revealed that treatment with the neurosteroids ALLO and PROG can reduce the aggregation of mHTT in astrocytes by regulating the mTOR-dependent autophagy pathway [150]. In in vivo studies, progesterone treatment in the 3-nitropropionic acid (3-NP) mouse model attenuates oxidative stress and suppresses excessive activation of microglia and astrocytes, thereby protecting striatal neurons and significantly improving motor function [151].

## Disordered cholesterol metabolism in AD

The pathological hallmarks of AD are characterized by progressive neuronal loss, tightly linked to the accumulation of Aβ plaques and hyperphosphorylated tau neurofibrillary tangles [152, 153]. Dysregulation of cholesterol metabolic homeostasis has emerged as a pivotal etiological factor in AD pathogenesis. In the brains of AD patients, while free cholesterol levels remain relatively stable, lanosterol levels are significantly reduced [154]. Notably, cerebral levels of 24S-OHC are decreased [154, 155], in contrast to elevated levels of 27-OHC [155, 156]. Also, the expression of DHCR24 and CYP46A1 is downregulated [157, 158].

## Cholesterol metabolism regulates Aβ aggregation and clearance

Intracellular cholesterol alterations exert a profound influence on the biogenesis of Aβ. Cholesterol modulates Aβ production and degradation through direct interactions with γ-secretase, APP, and Aβ peptides. The processing of APP occurs at the membrane interface: α-secretase-mediated cleavage releases soluble extracellular fragment sAPPα, whereas BACE1 cleavage generates soluble extracellular fragment APPβ and the membrane-anchored C99 fragment. γ-secretase then cleaves C99 to produce Aβ peptides [159]. Cholesterol binds to the cholesterol-recognition motif within presenilin-1 (PS1), the catalytic subunit of γ-secretase, through specific interactions involving its sterol ring. This binding event enhances γ-secretase activity and promotes the production of the pathogenic Aβ42 isoform [160]. Similarly, cholesterol embeds into the cholesterol-binding domain of the transmembrane C99 fragment, the transmembrane domain of APP. This incorporation induces conformational bending of the APP, resulting in exposure of the BACE1 cleavage site, thereby facilitating Aβ generation [161]. Furthermore, cholesterol binds directly to specific regions within the Aβ peptide via van der Waals forces and hydrophobic interactions, triggering a conformational transition from random coil to β-sheet structure-thus accelerating Aβ oligomerization and fibrillization [162, 163].

**Fig. 2:**
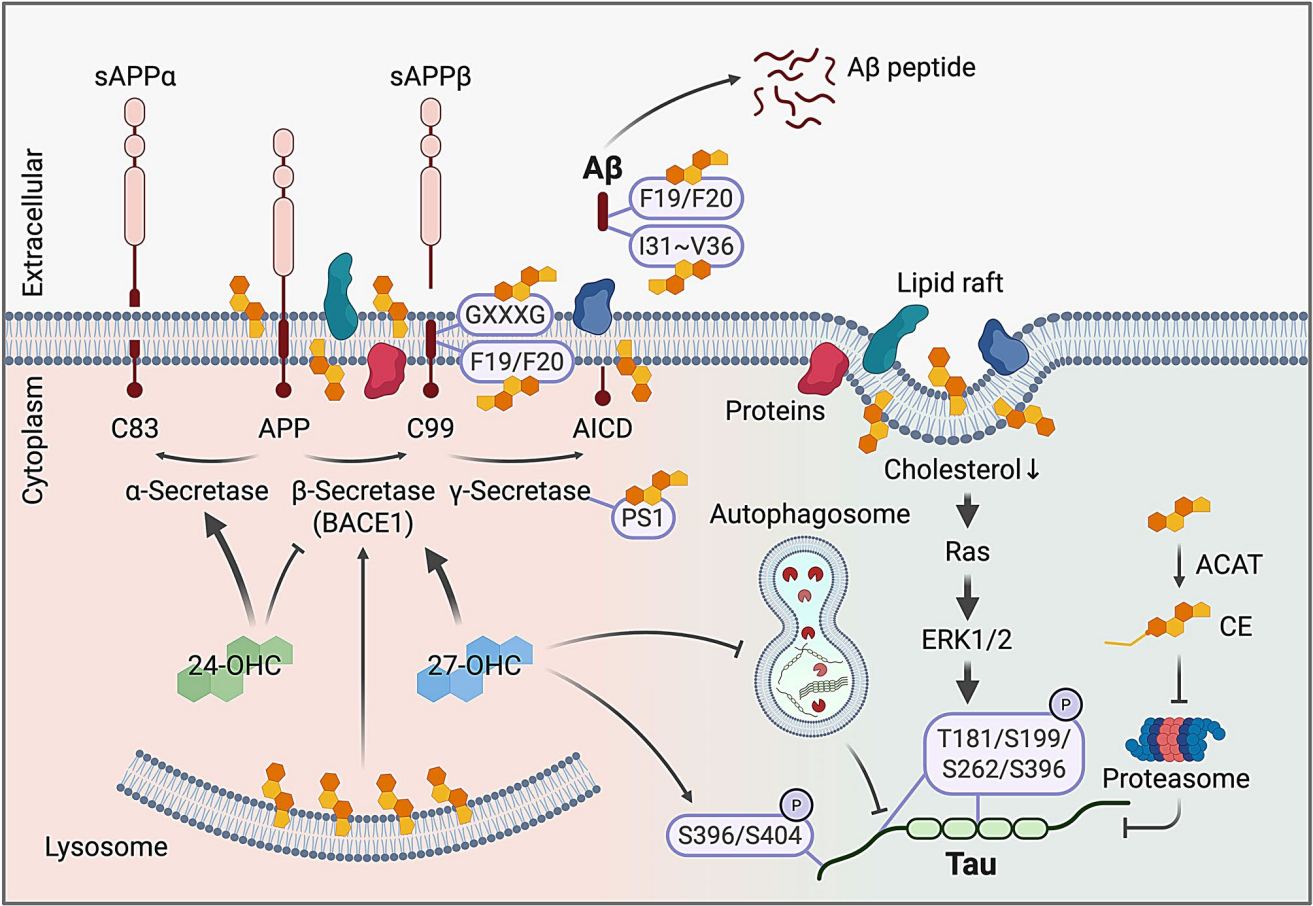
Cholesterol metabolism regulates Aβ generation and Tau phosphorylation. APP is cleaved by β-secretase cleavage to generate C99, which is subsequently processed by γ-secretase to form Aβ. Cholesterol-rich lipid rafts facilitate the interaction between APP and β-/γ-secretases. Cholesterol binds to the transmembrane region of C99, exposing the BACE1 cleavage site. It also interacts with Aβ, accelerating its oligomerization and fibrillation. Additionally, cholesterol binds to the core catalytic subunit PS1 of γ-secretase, promoting the cleavage of APP to produce Aβ. 24S-OHC enhances α-secretase activity and inhibits BACE1, while 27-OHC promotes BACE1 activity and Aβ production. High cholesterol levels in lysosomes also facilitate BACE1 cleavage of APP and C99, leading to increased Aβ generation. Reduction of cholesterol in lipid rafts promotes Tau phosphorylation via the Ras and ERK1/2 pathways. Furthermore, 27-OHC promotes phosphorylation, while inhibiting the fusion of autophagosomes and lysosomes, impairing Tau degradation. Additionally, cholesterol is esterified by ACAT to form cholesterol esters, which inhibit proteasomal degradation of Tau. Phe, phenylalanine. Ile, isoleucine. Val, valine. Ser, serine. Thr, threonine. Thick arrows indicate pathways mainly supported by human patient-based evidence (including clinical cohort studies, CSF/blood sample analyses, post-mortem brain tissue studies of patients). Thin arrows indicate pathways only supported by preclinical models, including mouse disease models, cell models (neuronal/glial cell lines, primary cells), and cell-free biochemical systems. Created in https://BioRender.com

In addition to direct binding, cholesterol indirectly modulates Aβ biogenesis and degradation through four distinct mechanisms. First, cholesterol drives the formation of lipid rafts-cholesterol-rich microdomains within the membrane. Under hypercholesterolemic conditions, APOE stabilizes these microdomains, facilitating the translocation of APP into lipid rafts, where APP undergoes sequential cleavage by BACE1 and γ-secretase to generate amyloidogenic Aβ peptides. Conversely, under hypocholesterolemic conditions, APP dissociates from GM1-enriched lipid rafts, rendering it susceptible to α-secretase-mediated cleavage and resulting in the production of the non-amyloidogenic sAPPα [164, 165]. Second, cholesterol participates in vesicular trafficking to perturb APP transport dynamics. Depletion of cellular cholesterol inhibits APP trafficking from early endosomes to the trans-Golgi network (TGN). Knockdown of *Dhcr24*, which reduces intracellular cholesterol levels, may downregulate the expression of key vesicle transport-associated proteins such as flotillin-1 and sorting-related receptor 1 (SorLA). This leads to decreased APP accumulation in endosomes, impaired trafficking, and ultimately enhanced Aβ deposition [166]. Moreover, during APP trafficking, its metabolite C99 accumulates in the ER to trigger increased cellular cholesterol uptake [167]. Under hypercholesterolemic conditions, cholesterol enrichment in lysosomal membrane impairs autophagosome-lysosome fusion, resulting in the accumulation of APP and C99 in lysosomes for cleavage by BACE1 to promote Aβ production [168]. Third, HDL-mediated reverse cholesterol transport (RCT) facilitates cholesterol efflux from the brain parenchyma, while apolipoproteins (e.g., APOE) on HDL particles contribute to Aβ clearance. Compared to APOE3 and APOE2 preferentially binding HDL, APOE4 exhibits a higher binding affinity for VLDL, thereby diminishing its cholesterol transport efficacy [169, 170]. APOE4 not only impairs the integrity of endothelial tight junctions by reducing phosphorylated occludin levels [171] but also activates the CypA-MMP9 signaling pathway in CSF to promote BBB breakdown and Aβ plaque dissemination [172, 173]. Furthermore, upon interaction with APOE4, LilrB3-a microglial surface receptor activates interferon-stimulated genes (ISGs), and impairs microglial phagocytic capacity, which leads to increased Aβ deposition and AD susceptibility [174]. Fourth, cholesterol-derived metabolites contribute to the regulation of Aβ production and degradation. Inhibition of ACAT reduces the biosynthesis of CE and suppresses Aβ generation [175]. 24S-OHC promotes APP processing through the non-amyloidogenic α-secretase pathway and inhibits Aβ42 production. In contrast, 27-OHC increases the levels of APP and BACE1 to enhance Aβ production [99]. A high 27-OHC/24S-OHC ratio correlates with elevated Aβ production [176]. The schematic diagram is shown in Fig. 2.

## Cholesterol metabolism regulates Tau phosphorylation and clearance

Tau protein is a central driver in AD pathogenesis, with its abnormal post-translational modifications, aggregation, and propagation, contributing to neuronal injury and cognitive deterioration [177]. Tau’s phosphorylation such as p-tau181 and p-tau217 shows a positive correlation with AD neuropathology [178–181]. Aβ deposition acts as an upstream trigger for pathological Tau phosphorylation [182, 183], and phosphorylated Tau reciprocally enhances Aβ oligomerization [184].

Cholesterol modulates Tau phosphorylation and clearance through several mechanisms: alteration in lipid microenvironment, activation of signaling pathways, modulation of enzyme activity, and impairment of autophagy. First, cholesterol activates phosphorylation signaling pathways via disruption of lipid rafts. Reduced cholesterol levels disrupt lipid raft integrity, leading to activation of Ras proteins localized within rafts. This subsequently triggers the MEK/ERK phosphorylation cascade, ultimately promoting hyperphosphorylation of Tau protein at Thr181, Ser199, and Ser262 residues. In astrocytes, knockdown of *Dhcr24* decreases membrane cholesterol, directly activating the Ras/MEK/ERK pathway and elevating Tau phosphorylation levels [185]. Second, cholesterol oxidative metabolites impair Tau clearance by disrupting autophagy and lysosomal function. High-cholesterol diets upregulate *Cyp27a1* expression, increasing levels of oxidative metabolites, such as 27-OHC and 24S-OHC. These metabolites suppress key autophagy pathways (e.g., via mTOR overactivation), and upregulate Tau-phosphorylating enzymes (Gsk3β and Cdk5), thereby increasing p-Tau levels and contributing to cognitive deficits in mice [186]. Conversely, knockdown of *Cyp27a1* reduces 27-OHC levels with decreased p-Tau expression. Third, CEs modulate Tau phosphorylation and degradation. Fluctuations in neuronal CE levels are linked to p-Tau accumulation. Reducing CE levels increases both proteasome content and activity, thereby facilitating the proteasomal degradation of p-Tau. Additionally, CEs stimulate Aβ42 secretion through interactions with cholesterol-binding domain of APP [187].

The schematic diagram is shown in Fig. 2.

Advances in drugs targeting cholesterol metabolism for the treatment of AD are outlined in Table 2.

**Table 2:** Drugs targeting cholesterol metabolism for AD treatment

Drug (Targets)	Function
Obicetrapib [163] (CETP)	modulates lipid profiles; ameliorates APOE4-related cholesterol dysregulation [NCT06005597] [188]
DA1 (ATAD3A)	inhibits ATAD3A oligomerization to normalize brain cholesterol homeostasis; suppress AD-related pathology; improve cognitive impairment [158]
Fatostatin (SREBP)	lowers neuronal cholesterol levels; reduces the pThr231Tau/t-Tau ratio [187]
Statins (HMGCR)	blocks the mevalonate pathway; reduces CE accumulation in neurons; decreases p-Tau and Aβ [187].
Efavirenz (CYP46A1)	activates CYP46A1 and cerebral cholesterol turnover; reduces amyloid burden, microglial activation and APP protein levels [189].
LXR agonist (LXR)	enhances lipid efflux; mitigates tau pathology and neurodegeneration [190].
Methyl-β-cyclodextrin (cholesterol)	promotes cholesterol efflux; protects APOE4 cells from lipid-peroxide mediated death [191].
Glutathione ethylester (GSH)	reverses oxidative stress; clears OPTN aggregates; restores mitophagic function [168].
α-mangostin (LDLR)	enhances the Aβ-clearing capacity of microglia [192].
CI-1011 (ACAT)	regulates secretory trafficking of APP; decreases Aβ generation [193].

## Disordered cholesterol metabolism in PD

Abnormal aggregation of α-Synuclein (α-Syn) to form Lewy bodies, along with the degeneration and death of dopaminergic neurons in the substantia nigra, are the core pathological features of Parkinson’s disease [194]. α-Syn primarily expresses at the presynaptic membrane of neurons, and exhibits both high water solubility and a strong affinity for phospholipid membrane. In its membrane-bound state, α-Syn maintains neuronal homeostasis by regulating synaptic vesicle trafficking and neurotransmitter release [195]. In PD patients, α-Syn misfolding into β-sheet-rich oligomers and fibrils, which aggregate into Lewy bodies with disrupted calcium homeostasis and mitochondrial function to trigger dopaminergic neuronal damage [196, 197]. Additionally, α-Syn oligomers activate microglia and astrocytes via the NLRP3 inflammasome to release pro-inflammatory cytokines (e.g., IL-1β, TNF-α) and exacerbate neuroinflammation [198].

## Cholesterol metabolism regulates α-synuclein aggregation and spread

Cholesterol promotes α-Syn aggregation through two major mechanisms: direct physical binding and chemical modification by oxidative metabolites. α-Syn contains distinct cholesterol-binding domains. Cholesterol binding attenuates electrostatic and hydrophobic interactions between α-Syn and lipid membranes, inducing a conformational shift toward β-sheet structures and accelerating the formation of toxic fibril [199]. Furthermore, cholesterol-rich lipid rafts serve as platforms for α-Syn aggregation [200, 201]. Cholesterol binding is dependent on specific lipid environments [202]. In DOPC-enriched membranes, cholesterol enhances the β-sheet propensity of the NAC region via hydrogen bonding, thereby promoting α-Syn aggregation. In contrast, in membranes rich in DOPE and DOPG, cholesterol inhibits the membrane insertion of the N-terminal and C-terminal domains by reducing membrane fluidity and minimizing lipid defects, which in turn hinders α-Syn oligomerization [199, 203]. In PD, elevated HMGCR activity and downregulated ABCA1 transporter function drive intracellular cholesterol accumulation [19, 51]. Pharmacological inhibition of cholesterol synthesis (e.g., statins) reduces α-Syn aggregation in neurons, whereas exogenous cholesterol supplementation exacerbates this process [199, 204].

In Lewy bodies from PD patients, over 90% of α-Syn is phosphorylated at Ser129, which represents one of the most prominent pathological features of PD [205]. Phosphorylation alters the interaction between the N-terminus and C-terminus of α-Syn by introducing negative charges, disrupting its native conformation and promoting the formation of β-sheet structures. In lipid raft microdomains, cholesterol enrichment enhances the accessibility of casein kinase 2 (CK2) to the Ser129 site of α-Syn, promoting Ser129 phosphorylation. Meanwhile, cholesterol inhibits the activity of Protein Phosphatase 2A (PP2A), reducing the dephosphorylation of Ser129 and facilitating the aggregation of α-Synuclein [206, 207]. Small molecule oxidation products derived from cholesterol can also accelerate the mis-assembly of α-Syn [208]. For example, oxidized cholesterol derivatives (e.g. 7-KC) covalently modify cysteine residues of α-Syn, enhancing its aggregation potential and neurotoxicity. The high oxidizing activity of 7-KC enables direct reactions with lysine residues, forming stable covalent crosslinks that lock α-Syn in aggregation-prone conformations [209–211].

**Fig. 3:**
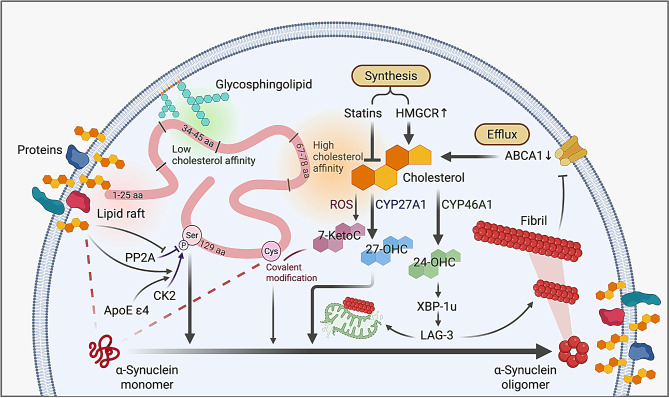
Cholesterol metabolism regulates α-synuclein aggregation and spread. In PD brains, elevated HMGCR and reduced ABCA1 cause cholesterol accumulation; statins lower intracellular cholesterol. Cholesterol oxidation to 7-KC promotes α-syn aggregation. 27-OHC facilitates α-syn spread, while 24-OHC mediates mitochondrial dysfunction and α-synuclein spread via XBP-1 and LAG-3. Cholesterol-rich lipid rafts platform α-syn aggregation, and α-syn fibrils inhibit ABCA1. Cholesterol and APOE ε4 enhance CK2 phosphorylation of α-synuclein, and cholesterol further inhibits PP2A-mediated dephosphorylation, promoting α-syn aggregation. The grading principle for arrow thickness is consistent with that in Fig. 2. Created in https://BioRender.com

Notably, 27-OHC promotes mitochondrial localization of α-Syn fibrils, amplifying pathological spread and dopaminergic neurodegeneration. Genetic ablation of *Cyp27a1* (the enzyme mediating 27-OHC synthesis) mitigates α-Syn pathology propagation [114, 212]. Elevated *Cyp46a1* and 24-OHC have been shown to promote the spread of α-Syn, whereas genetic removal of *Cyp46a1* attenuates α-Syn-induced neurotoxicity [213]. Oxidized cholesterol products can also promote the abnormal aggregation of α-Syn by altering the physicochemical properties of cell membranes, such as reducing fluidity and disrupting lipid packing [214, 215]. In PD patients, the level of APOE4 in CSF is significantly positively correlated with the concentration of pS129-αSyn. APOE4 binds to the N-terminal non-amyloid component (NAC region) of α-Syn through lipid raft microdomains, inducing its conformational transition from α-helix to β-sheet [216]. Meanwhile, APOE4 enhance the phosphorylation of α-Syn at the Ser129 site by activating CK2 [217]. Additionally, cholesterol modulates α-Syn-induced synaptic vesicle clustering by lowering the local affinity within its non-amyloid-β component (residues 65–97), thereby enhancing vesicle-vesicle interactions and altering neurotransmitter release [218, 219]. Conversely, certain lipid molecules exert protective effects against α-Syn aggregation. For example, Lysophosphatidylcholine (LPC), a class of lipid molecules, selectively binds to monomeric α-Syn and promotes its transition from a relatively unstructured state to a compact and stable α-helical conformation, thereby inhibiting pathological aggregation [220]. Mechanistically, α-Syn resembles apolipoproteins and lipid-binding proteins, acting as a lipid receptor to mediate neuronal cholesterol efflux. However, when α-Syn aggregates, it disrupts this function by inhibiting cholesterol efflux transporters such as ABCA1, thereby exacerbating intracellular cholesterol accumulation [221]. The schematic diagram is shown in Fig. 3.

Advances in drugs targeting cholesterol metabolism for the treatment of PD are outlined in Table 3.

**Table 3:** Drugs targeting cholesterol metabolism for PD therapy

Drug (Targets)	Function
Simvastatin (HMGCR)	inhibits microglial activation and inflammation; reduces α-syn aggregation; preserves DA neurons; reduces dementia and PD incidence [222–225].
Atorvastatin (HMGCR)	stabilizes tight junctions; enhances neurite outgrowth via Akt/GSK-3β; reduces neuroinflammation [226–229]
Probucol (ABCA1)	inhibits microglial M1 polarization; protects mitochondria; alleviates oxidative stress [230–233]
Sodium phenylbutyrate (SQS)	inhibits microglial activation and neuroinflammation; reduces α-syn accumulation and improves motor function [234–236]
Ezetimibe (NPC1L1)	suppresses microglial activation; exerts neuroprotective effects via AMPK/SIRT-1/PGC-1α; promotes autophagy; reduces apoptosis [237–239]
Efavirenz (CYP46A1)	mitigates α-syn propagation and neuroinflammation; improves behavioral changes [240, 241]
GW3965 (LXR)	protects DA neurons and striatal-projecting fibers; inhibits microglial and astroglial activation [242, 243]
α-mangostin (HMGCR, NPC1L1, LXR)	activates cAMP/PKA pathway; accelerates degradation of α-syn in a UPS dependent manner [244]

## Disordered cholesterol metabolism in HD

HD is an autosomal dominant neurodegenerative disorder primarily characterized by psychiatric manifestations, progressive cognitive decline, and choreiform movements. The causative mutation (an expanded CAG trinucleotide repeat in the HTT gene) promotes mHTT misfolding, aggregation, and sequential neuronal dysfunction and loss [245, 246]. Dysregulated cholesterol metabolism has emerged as a prominent pathological feature of HD. Clinical studies suggest that de novo cholesterol synthesis is downregulated in HD patients, as reflected by decreased expression of cholesterol biosynthetic genes and reduced plasma levels of 24(S)-OHC, which correlate positively with disease severity and progression. Furthermore, advanced-stage HD patients exhibit significantly reduced levels of total cholesterol, LDL, HDL, and cholesterol biosynthetic precursors [246]. HD mouse models recapitulate these abnormalities, showing reduced expression of critical cerebral cholesterol synthesis and oxidation genes (*Hmgcr*, *Cyp51*, *Dhcr7*, and *Cyp46A1*) [31, 116], as well as decreased cerebral and systemic levels of cholesterol, desmosterol, 24(S)-OHC, and cholesterol biosynthetic intermediates [247].

## Cholesterol metabolism regulates the accumulation and clearance of HTT aggregates

The N-terminal 17-amino acid region (Nt17) of huntingtin (HTT), localized within its CAG repeat expansion domain, adopts an amphipathic α-helical conformation. This structural hallmark enables extensive intermolecular interactions, facilitating oligomerization and interactions with association with diverse lipid membranes [248]. Consequently, HTT localizes to and associates with multiple membrane compartments, including the plasma membrane [249], endoplasmic reticulum [250], and mitochondria [251]. Importantly, cholesterol levels modulate the affinity of HTT-lipid membrane interactions and the aggregation propensity of HTT, with these regulatory effects exhibiting membrane system-specificity dependent on distinct lipid compositions. In membranes composed of 1,2-dioleoyl-sn-glycero-3-phosphocholine (DOPC), increased cholesterol expands membrane surface area and induces membrane defects, thereby enhancing HTT membrane binding, promoting fibril formation, and facilitating assembly of HTT trimer- and tetramer-lipid complexes. In contrast, membranes composed of 1-palmitoyl-2-oleoyl-sn-glycero-3-phosphoglycerol (POPG) form tightly packed bilayers via intermolecular connections and hydrogen bonding, which mitigates electrostatic repulsion between lipid head groups. Cholesterol disperses lipid head groups, increases HTT binding sites, and promotes formation of HTT dimer-lipid complexes. Conversely, in 1-palmitoyl-2-oleoyl-sn-glycero-3-phosphocholine (POPC) membranes, exogenous cholesterol reduces membrane fluidity and diminishes membrane defects, thereby attenuating HTT-lipid membrane interactions and reducing HTT aggregation [252]. Intrastriatal infusion of cholesterol via osmotic minipumps in HD mice ameliorates motor deficits and prevents cognitive decline in a dose-dependent manner. This intervention also enhances endogenous cholesterol biosynthesis, facilitates clearance of mutant HTT (mHTT), and restores neuronal morphology and function [253]. Furthermore, systemic administration of peptide-modified, cholesterol-loaded nanoparticles via intraperitoneal injection in an HD mouse model rescues cognitive deficits, alleviates behavioral impairments, and reduces mHTT aggregation in the striatum and cerebral cortex [254]. Collectively, these findings demonstrate that the cholesterol-based interventions confer therapeutic benefit in both early and late stages of HD.

**Fig. 4:**
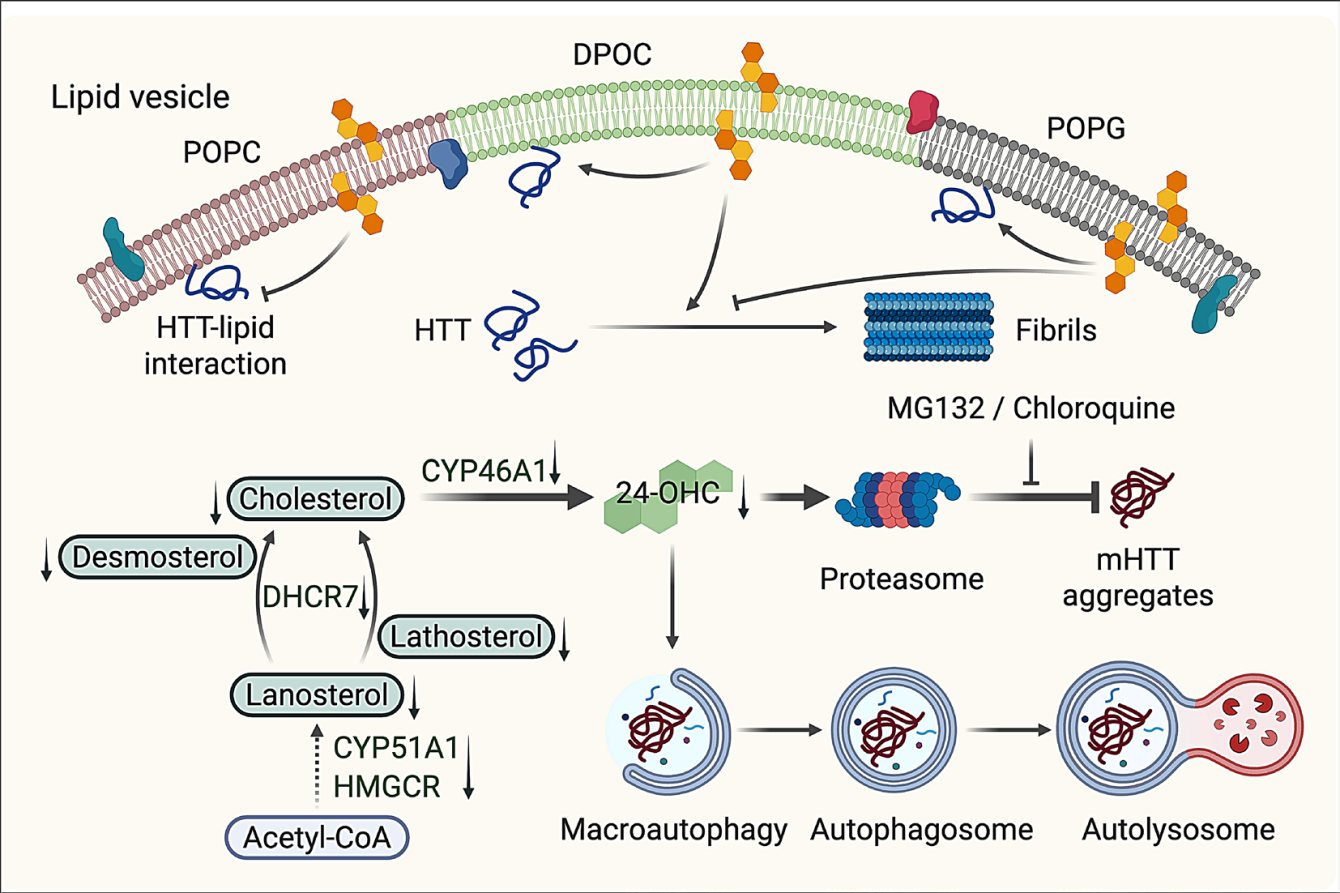
Cholesterol metabolism regulates HTT aggregation and clearance. In HD, the expression levels of cholesterol synthesis-related genes are downregulated, leading to decreased levels of cholesterol precursors, cholesterol, and 24S-OHC. In artificial membrane systems, cholesterol can regulate the formation of HTT-lipid complexes and HTT aggregates. Furthermore, intracellular overexpression of CYP46A1 promotes the upregulation of proteasome-related gene expression, enhances the recognition and phagocytosis of mHTT aggregates by autophagosomes, thereby effectively reducing the accumulation of mHTT aggregates in the cytoplasm. The grading principle for arrow thickness is consistent with that in Fig. 2. Created in https://BioRender.com

Accumulating evidence indicates that cholesterol CYP46A1 facilitates the clearance of mHTT aggregates. In striatal neurons expressing the first exon of HTT with an expanded CAG repeat tract, CYP46A1 overexpression restores cholesterol metabolism disrupted by the accumulation of toxic mHTT species, and markedly attenuates mHTT aggregation via upregulating proteasome expression [116]. Furthermore, cholesterol biosynthetic precursors, including desmosterol, ergosterol, and lathosterol, exhibit analogous inhibitory effects on mHTT aggregation [255]. Autophagic impairment is a key pathological hallmark of HD, and defective autophagosome cargo in HD patient-derived cells, HD mouse models, or in vitro HD cellular models could result in inefficient sequestration of intracellular substrate [256]. In zQ175 HD model mice, CYP46A1 overexpression enhances autophagosome capacity to capture protein substrates, predominantly mHTT-related abnormal proteins. This intervention promotes autophagosome-lysosome fusion and subsequent cargo degradation, improves cellular clearance of misfolded proteins, ultimately resulting in a significant reduction in mHTT levels [255]. Collectively, these findings indicate that CYP46A1 exerts potent neuroprotective effects by dually regulating mHTT clearance through coordinated activation of the proteasomal and autophagic pathways. The schematic diagram is shown in Fig. 4.

Advances in drugs targeting cholesterol metabolism for the treatment of HD are outlined in Table 4.

**Table 4:** Drugs targeting cholesterol metabolism for HD treatment

Drug (Targets)	Function
Simvastatin (HMGCR)	upregulates Bcl-2 activity; downregulates Bax activity; attenuates neuronal loss [33]
Atorvastatin (HMGCR)	mitigates oxidative stress and neuroinflammation; ameliorates motor deficits [34]
Cholesterol	reduces mHTT aggregation; restores synaptic transmission function; ameliorates cognitive impairments and behavioral deficits [253, 254]
AB1001 (CYP46A1)	reduces mHTT aggregation; attenuates striatal neuronal atrophy and motor deficits [116]
T0901317 (LXR agonist)	suppresses amplitude of sIPSCs [257]; partly ameliorates early developmental deficits [54]
Ketogenic Diet (KD)	ameliorates motor deficits; delays weight loss; reduces MSNs electrophysiological abnormalities [257]

## Perspective

Emerging evidence indicates the pivotal role of cholesterol metabolism in the pathogenesis and therapeutic strategies for AD, PD, and HD. Currently, this field contains several research limitations: autopsy-derived brain tissue data only reflect end-stage pathological changes; most mechanistic studies rely on transgenic mouse or toxin-induced models, which fail to adequately recapitulate the complexity of AD/PD/HD in human patients; insufficient attention about sex-specific differences in cholesterol metabolism; the interplay between cholesterol metabolism and inflammation, oxidative stress, mitochondrial dysfunction remains incompletely elucidated; single-target drug is difficult to cover multiple pathological pathways. Given that early intervention is superior, it is important to identify pathogenic clues before symptom onset. Screening high-risk populations based on biomarkers such as APOE genotypes and 7β-hydroxycholesterol will facilitate precise intervention such as dietary guidelines for those populations. For future research, it is critical to use integrative approach, including single-cell sequencing, spatial transcriptomics, and high-throughput metabolomics, to delineate the spatiotemporal dynamics of cholesterol metabolism during the onset/progression of AD, PD, and HD. Systemic and interdisciplinary investigations might also provide clues about multi-targets combined therapeutic strategies. In addition, designing carriers such as lipid nanoparticles to enhance drug delivery specifically into the central nervous system) is crucial, which helps to reduce side effects in other organs.

## Rules of nomenclature

In this review, the nomenclature rules for gene and protein names are as follows:

Human: genes are written in all uppercase letters and italicized; proteins are written in all uppercase letters and roman (non-italicized). Mouse: genes are written with the first letter uppercase, the remaining letters lowercase, and italicized; proteins are written with the first letter uppercase, the remaining letters lowercase, and roman (non-italicized).

## Data Availability

Not applicable.

## Abbreviations

Aβ: Amyloid-β
ABCA1: ATP-Binding Cassette Transporter A1
ABCG1: ATP-Binding Cassette Transporter G1
Acat1/Soat1: Cholesterol acyltransferase 1
AD: Alzheimer’s disease
AICD: Amyloid intracellular domain
ALLO: Allopregnanolone
ApoA-I: Apolipoprotein A-I
APOE: Apolipoprotein E
APP: Amyloid precursor protein
BACE1: β-site amyloid precursor protein cleaving enzyme 1/beta-secretase 1
BBB: Blood-Brain Barrier
BDNF: Brain-derived neurotrophic factor
C83/C99: Carboxy-terminal fragment 83/99
CETP: Cholesteryl ester transfer protein
Ch25h: 25-hydroxylase
CNS: Central nervous system
COPII: Coat Protein Complex II
CREB: cAMP response element-binding protein
CYP27A1: Cholesterol 27-hydroxylase
Cyp46a1: Cholesterol 24-hydroxylase
CK2: Casein kinase
DAM: Disease-associated microglia
5α-DHP: 5α-dihydroprogesterone
DHEAS: Dehydroepiandrosterone sulfate
DMAPP: Dimethylallyl pyrophosphate
5α,6α-EC: 5α,6α-epoxycholesterol
ER: endoplasmic reticulum
FAs: Fatty acids
ERK1/2: Extracellular signal-regulated kinase 1/2
FPP: Farnesyl pyrophosphate
HDL: High-density lipoproteins
Hipp: Hippocampus
HMG-CoA: 3-hydroxy-3-methylglutaryl-coenzyme A
HMGCR: 3-hydroxy-3-methylglutaryl-CoA reductase
HTT: Huntingtin
Indip: Insig-1-derived interfering peptide
INSCs: Induced neural stem cell lines
IPP: Isopentenyl pyrophosphate
IRβ: Insulin receptor β
7-KC: 7-ketocholesterol
LAG3: Lymphocyte-activation gene 3
LCAT: Lecithincholesterol acyltransferase
LDL: Low-density lipoproteins
L-DOPA: Levodopa, L-3,4-dihydroxyphenylalanine
LDs: Lipid droplets
LNPs: Lipid nanoparticles
LPC: Lysophosphatidylcholine
LPS: Lipopolysaccharide
LRP1: Low-density lipoprotein receptor-related protein-1
LSS: Lanosterol synthase
LUNAR: Light-Using Nanoscale Augmented Raman
LXR: Liver X receptor
MβCD: Methyl-β-cyclodextrin
mHTT: Mutant HTT
miRNAs: MicroRNAs
MPTP: 1-methyl-4-phenyl-1,2,3,6-tetrahydropyridine
MPP^+^: 1-methyl-4-phenylpyridinium
mTORC1: Mechanistic Target of Rapamycin Complex 1
MVA: Mevalonic acid
NCEH1: Neutral cholesterol ester hydrolase
NLRP3: NOD-Like Receptor Family, Pyrin Domain-Containing 3
NMDAR: N-methyl-D-aspartate glutamate receptor
NPC1L1: Niemann-Pick C1-like 1
Nrf-2: Nuclear Factor Erythroid 2-Related Factor 2
6-OHDA: 6-hydroxydopamine
24-OHC: 24-hydroxycholesterol
25-OHC: 25-hydroxycholesterol
27-OHC: 27-hydroxycholesterol
OPCs: Oligodendrocyte progenitor cells
PCSK9: Proprotein convertase subtilisin/kexin type 9
PD: Parkinson’s disease
PGC1α: Peroxisome Proliferator-Activated Receptor Gamma Coactivator 1-Alpha
PGE2: Prostaglandin E2
PPARα: Peroxisome proliferator-activated receptor alpha
PP2A: Protein phosphatase
PREG: Pregnenolone
PREGS: Pregnenolone sulfate
PROG: Progesterone
PTMs: Post-translational modifications
ROS: Reactive oxygen species
sAPPα/β: Soluble amyloid precursor protein alpha/beta
SCAP: SREBP cleavage-activating protein
SIRT1: Sirtuin 17α/7β-OHC7α/7β-hydroxycholesterol
SNAP-25: Synaptosomal-Associated Protein 25 kDa
SOAT1: Sterol O-acyltransferase 1
SREBPs: Sterol regulatory element-binding proteins
SQS: Squalene synthase
SSD: Sterol-sensing domain
α-Syn: α-Synuclein
TLR4: Tolllike receptor 4
TREM2: Triggering receptors expressed on myeloid cells 2
USP20: Ubiquitin-Specific Peptidase 20
VLDLR: Very Low-Density Lipoprotein Receptor
XBP1: X-box binding protein 1
